# Development of an Oral Form of Azacytidine: 2′3′5′Triacetyl-5-Azacytidine

**DOI:** 10.1155/2011/965826

**Published:** 2011-12-18

**Authors:** Amy Ziemba, Eugene Hayes, Burgess B. Freeman, Tao Ye, Giuseppe Pizzorno

**Affiliations:** ^1^Nevada Cancer Institute, One Breakthough Way, Las Vegas, NV 89135, USA; ^2^St. Jude Children's Research Hospital, Memphis, TN 38105, USA; ^3^The Hong Kong Polytechnic University, Kowloon, Hong Kong

## Abstract

Myelodysplastic syndromes (MDSs) represent a group of incurable stem-cell malignancies which are predominantly treated by supportive care. Epigenetic silencing through promoter methylation of a number of genes is present in poor-risk subtypes of MDS and often predicts transformation to acute myelogenous leukemia (AML). Azacitidine and decitabine, two FDA-approved DNA methyltransferase (DNMT) inhibitors, are able to improve overall response although their oral bioavailability complicates their clinical use. This study evaluated 2′, 3′, 5′-triacetyl-5-azacitidine (TAC) as a potential prodrug for azacitidine. The prodrug demonstrated significant pharmacokinetic improvements in bioavailability, solubility, and stability over the parent compound. In vivo analyses indicated a lack of general toxicity coupled with significantly improved survival. Pharmacodynamic analyses confirmed its ability to suppress global methylation in vivo. These data indicate that esterified nucleoside derivatives may be effective prodrugs for azacitidine and encourages further investigation of TAC into its metabolism, activity, and possible clinical evaluation.

## 1. Introduction

Currently, it is estimated that between 12,000 and 20,000 new cases of MDS are diagnosed each year in the United States. Although MDS can affect all ages, the highest prevalence occurs in those over 60 years of age [1, 2]. Much of the population has indolent forms of MDS, making it one of the most prevalent hematologic malignancies of older adults.

MDS represents a heterogeneous group of hematopoietic disorders which are derived from an abnormal multipotent progenitor cell and are characterized by hyperproliferative bone marrow, cellular dysplasia, and ineffective hematopoiesis [3]. Morbidity and mortality result from anemia, bleeding, and infection, along with transformation to acute myelogenous leukemia (AML) in approximately one-third of patients [4, 5]. The basis of therapy is supportive care, including red blood cell or platelet transfusions and treatment of infections. Stem-cell transplantation remains the only chance for cure, but it is associated with significant treatment-related morbidity and mortality and is generally restricted to patients <60 years of age [6]. Similar limitations exist for the use of high dose chemotherapy. Given the limitations of existing therapies, there is a clear need for additional therapeutic options for patients with MDS.

Cancer cells are characterized by abnormal DNA methylation patterns, and DNA hypermethylation is suspected of being involved in MDS progression and leukemogenesis. Therefore, inhibitors of DNA methylation represent a useful approach to revert these epigenetic changes. 5-azacitidine (Vidaza) and its derivative 5-aza-2′-deoxycytidine/decitabine (Dacogen) are nucleoside analogs with DNA hypomethylating activity that have been FDA-approved during the past 5-6 years for MDS treatment [7, 8]. They appear to induce re-expression of key tumor suppressor genes in MDS [9]. Compared to supportive care, both agents show an improved overall response (60% versus 5%), a longer time to progression to AML or death, but still with limited overall survival advantage [10]. Azacitidine has been developed for the treatment of acute leukemia and is currently being evaluated in a variety of other disorders [11].

DNA methylation results in the addition of a methyl group at the carbon 5 position of the cytosine ring in CpG dinucleotides, which is critical to chromatin structure and genomic stability [12]. Since 5-methyl cytosine can be deaminated to thymidine, DNA hypermethylation also facilitates gene mutations in human cancers [13]. The transferring of methyl groups from S-adenosylmethionine to cytosine is catalyzed by DNA methyltransferases, the best known of which is DNMT1. The inactivation of DNMTs has been shown to be the most effective method of inhibiting DNA methylation although it is recognized that this approach lacks specificity. However, inhibiting the activity of DNMTs has resulted in the abrogation of tumorigenicity in murine cancer models [14]. 

DNMT-inhibiting nucleoside analogs require metabolism by kinases into nucleotides before their incorporation into DNA and/or RNA in order to inhibit DNA methylation. The modification at C5 prevents the release of DNMTs by forming a covalent complex, preventing further DNA methylation and consequently the DNA of the progeny cells is not methylated [15]. Azacitidine and Decitabine are extremely potent in inhibiting DNA methylation, but their short half-lives in aqueous solutions [16, 17] and low oral bioavailability complicate their delivery. For years, these drugs used as antineoplastic agents in leukemias were escalated to maximum tolerated doses (MTD) [18]; however, recent clinical trials have confirmed that low-dose exposures lead to greater responses and are associated with less toxicity [19, 20].

In an effort to overcome the stability and pharmacokinetic limitations, a number of groups are working to develop oral DNMT inhibitors, including oral forms of decitabine and azacitidine. As a proof of principle we have synthesized an acetylated derivative of azacitidine, 2′,3′,5′-triacetyl-5-azacitidine (TAC) to evaluate as an oral prodrug. While this is not a totally new approach, it was never utilized for this derivative and in this set of diseases. Our current study demonstrates that 2′,3′,5′-triacetyl-5-azacitidine (TAC) effectively inhibits methylation and improves life expectancies in murine models while demonstrating lower toxicity when compared to its parent compound, azacitidine.

## 2. Materials and Methods

### 2.1. Preparation of 2′,3′,5′-Triacetyl-5-Azacitidine (TAC) [21]

TAC was prepared through condensation of trimethylsilylated-5-azacytosine and 1,2,3,5-tetra-O-acetyl-*β*-D-ribofuranose. In a 150 mL, 3-necked flask, a mixture of 5-azacytosine, hexamethyldisilazane, and ammonium sulfate was heated at reflux for 2 hours. A fresh amount of ammonium sulfate was added, and the reflux was continued for 6 hours. The excess hexamethyldisilazane was removed under reduced vacuum to afford trimethylsilylated 5-azacytosine as an off-white residue.

Tetra-O-acetyl-D-ribofuranose was prepared by adding acetic anhydride dropwise to D-ribose dissolved in dry pyridine. The solvent was removed by heating the solution at 50°C for 15 minutes, and tetra-O-acetyl-D-ribofuranose was crystallized from isopropanol [22].

Trimethylsilylated 5-azacytosine and 1,2,3,5-Tetra-O-acetyl-*β*-D-ribofuranose were dissolved in acetonitrile, and the mixture was cooled at 0°C in an ice-water bath. Trimethylsilyl trifluoromethanesulfonate (TMSOTf) was added slowly at 0°C then stirred at room temperature for 3 hours. The reaction mixture was poured into a solution of Na_2_CO_3_ and NaHCO_3_ in ice water and then extracted with dichloromethane. The combined organic layer was washed with cold H_2_O, cold brine, and dried over anhydrous Na_2_SO_4_. The residue, after removal of the solvent, was recrystallized from a mixture of dichloromethane and hexane to provide the desired compound with an 85% yield.

### 2.2. LC/MS/MS Analysis of TAC and Its Metabolites Mono- and Di-Acetyl-5-Azacitidine and ACT

To monitor the in vivo absorption, distribution, and metabolism of TAC, a highly sensitive LC/MS/MS analytical method was developed that allows the quantitation of concentrations as low as 10 ng/mL of TAC and its metabolites. The samples were prepared by adding 200 *μ*L of plasma to 400 *μ*L of cold acetonitrile, vortexed, and centrifuged at 14,000 rpm for 10 minutes. The supernatant was placed into a 10 × 75 mm glass test tube and evaporated to 50 *μ*L under nitrogen at 37°C. The residue was then diluted with 1 mL of 2% phosphoric acid and vortexed for 10 seconds at 3,000 rpm.

Solid phase extraction was conducted using a Varian Bond Elute PLEXA PCX 1 mL columns placed on a vacuum manifold. Columns were activated by adding 1 mL of MeOH followed by 1 mL H_2_O and 1 mL of 2% phosphoric acid. All samples were allowed to drip by gravity flow. The extraction columns were then washed sequentially with 1 mL 2% phosphoric acid, 1 mL H_2_O, and 1 mL MeOH/AcCN (1/1) and eluted into a 10 × 75 mm glass test tube containing 50 *μ*L of 25% formic acid in MeOH with 2 × 600 *μ*L of 2% NH_4_OH in MeOH/AcCN (1/1). The liquid was then evaporated to dryness with nitrogen at 37°C, and 100 *μ*L of mobile phase A was added and vortexed for 10 seconds at 3,000 rpm and transferred to LC autosampler vials at 4°C.

HPLC separation was conducted on a reverse phase C18 column (Varian Pursuit C18, 3 micron particle size; 100 mm × 2.0 mm) using a Shimadzu SIL-HTc controller with dual LC-20AD pumps and DGU-20A3 degasser. The separation was achieved using a gradient of Mobile Phase A: 0.1% Formic Acid and Mobile Phase B: 0.1% Formic Acid, 90% MeOH with a flow rate of 400 *μ*L/min at 40°C over 10 minutes. MS/MS fragmentation was performed on an Applied Biosystems API 3200 instrument with a Turbo V Ion Spray ion source and Positive ion polarity.

### 2.3. Drug Stability

ACT and TAC stabilities were determined after incubation at pH1 (2% Phosphoric acid), pH3 (0.1% Formic Acid), pH5 (K_3_PO_4_), and pH7.4 (Dulbecco's PBS). 100 ng of each compound was resuspended in 1.2 mL total volume, and 200 *μ*L samples taken at 1, 3, 6, 29, and 48 hr. Each pH point was repeated 6 times in 2 dram vials with Teflon-lined screw caps at room temperature. Samples were extracted by solid-phase extraction using 100 ng decitabine as an internal standard and analyzed by tandem mass spectroscopy as described above.

### 2.4. Log P Determination

The OSIRIS Property Explorer software was used to generate initial cLog*P* values. The partition coefficient was determined by using an analyte concentration near the solubility limit for the compound in water and incubating in a water : octanol mix ranging from 0.5 : 1, 1 : 1, to 2 : 1. The pH of the water was 2 units above the pKa of the compound, preventing ionization. The experiment was conducted using the shake-flask method, after allowing octanol and water to equilibrate for 24 hours at 25°C. The initial drug concentration in water was 0.2 mM. Tubes were rotated at a 45 degree angle for 10 minutes with a 3 second rotation. The test was performed in a constant environment of 20°C–25°C. Tubes were centrifuged at 1000 g for 10 minutes, and the octanol phase was extracted. The UV/Visible absorption spectrum was evaluated from 550 nm to 210 nm to determine the absorption maximum. The analyte concentration was determined and the Log*P* calculated: Log = log(concentration in octanol/concentration in water).

### 2.5. Permeability across Caco-2 Monolayers

Caco-2 cells (ATCC) were maintained under standard conditions in Dulbecco's modified Eagle's medium (DMEM). Approximately 1 × 10^5^ cells (passage number 25–35) were seeded onto polycarbonate cell culture inserts (Transwell, 0.45 *μ*m, 12 mm diameter). The cells were allowed to grow and differentiate for three weeks changing the medium every 2-3 days. Lucifer yellow was used to ensure appropriate paracellular permeability levels [23].

The transport experiments were performed in Hanks' balanced salt solution (HBSS) containing 25 mM Hepes (pH 7.4) as thoroughly described [24]. The drugs were dissolved in transport buffer to a final concentration of 0.2 mM. The monolayers were washed in prewarmed transport buffer for 30 min. The drug solutions were added to the donor side of the monolayers, and fresh transport buffer was added to the receiver side. The plates were gently shaken (50–100 rpm) at 37°C. At time intervals ranging from 5 minutes up to 2 h, 0.2 mL samples were collected from the receiver chambers and replenished with fresh transport buffer. Samples containing the transported drug was collected for LC/MS analysis by immediately freezing at −80°C and subsequently extracted 2 : 1 with acetonitrile and analyzed as indicated in the LC/MS/MS methodology section.

### 2.6. Pharmacokinetics and Metabolism of TAC in Mice

To evaluate the in vivo pharmacokinetic properties of TAC, C57/BL6 female mice of 20 g body weight were dosed with 38 mg/kg TAC p.o. (equimolar dose to 25 mg/kg of ACT) or 25 mg/kg ACT i.v. via tail vein injection. TAC and ACT were solubilized in 1X PBS immediately before administration and dosed at 0.01 mL/g fasted body weight. Blood samples (two time points per animal, three animals per time point) were obtained via retro-orbital bleeding with Natelson pipettes at 0.5, 1, 2, 4, 8, and 24 hours after drug administration. The collected blood was then centrifuged for 4 minutes at 13,000 g. Plasma was immediately extracted with cold acetonitrile and stored at −80°C until LC/MS/MS analysis.

Noncompartmental plasma pharmacokinetic parameters for TAC prodrug and ACT after oral TAC or i.v ACT administrations were estimated using the sparse sampling option in WinNonlin Software version 4.1 (Pharsight Corp., Mountain View, Calif, USA). Briefly, the WinNonlin sparse sampling option applies the linear trapezoidal rule for area under the concentration-time curve (AUC) calculations and generates a standard error for the mean AUC estimate that accounts for correlations in the data resulting from repeated sampling of individual mice. Parameters of interest included the AUC from time 0 to the last measurable concentration (AUC_last_), observed peak concentration after oral administration (C_max⁡_), estimated peak concentration after i.v. administration (C_0_), mean residence time to the last measurable concentration (MRT_last_), and the half-life of the terminal phase (T_1/2_). To assess the multiphasic decline in plasma concentrations, an alpha (T_1/2*α*_) half-life was also estimated using a log-linear regression of the mean ACT concentrations through 2 hours after dosing.

### 2.7. Global Methylation Detection Assay

24 hours following drug administration, snap-frozen mouse tissues were homogenized and genomic DNA isolated using Promega's Wizard Genomic DNA Purification Kit. 100 ng of EcoRI linearized genomic DNA was spotted onto PVDF membrane. The membrane was air-dried and repermeabilized according to manufacturer's instructions. Nonspecific binding was blocked using 5% milk in PBS-T and washed twice in PBS-T for 5 minutes. The membrane was incubated with primary antibody (1 : 1000 anti-5-methylcytosine in PBS-T, Calbiochem) for 1 hour then washed four times for five minutes each with PBS-T. The membrane was incubated with the HRP-conjugated secondary antibody for 1 hour and washed four times for five minutes each with PBS-T. Signal intensity following ECL detection was quantitated using KODAK Image Station software. Assay results were confirmed using EpiGentek's Methylamp Global DNA Methylation Quantification Kit per manufacturer's instructions.

### 2.8. p15INK4B Methylation Status

Mouse leukemia cells (L1210) were purchased from ATCC. The published IC50 value for ACT (0.2 uM) was first confirmed using the Promega CellTiter MTS assay. Cells were treated with ACT (0.1 or 1 uM) or TAC (1 or 100 uM) for 48 hours, with fresh drug added daily. Genomic DNA was isolated after 48 hours using the Sigma GenElute Genomic DNA Kit, including the RNase treatment step to ensure no RNA carryover. The methylation status of p15INK4B was determined using a predesigned primer set and the EpiTect Methyl qPCR Assays MethylScreen technology (Qiagen, Frederick, Md, USA).

### 2.9. Antileukemic Activity of Triacetyl-5-Azacitidine

The in vivo L1210 lymphocytic leukemia model in C57BL/6 × DBA/2 F1(BDF1) female mice was used to evaluate the in vivo antiproliferative effect of TAC. L1210 cells were carried in BDF1 mice by weekly i.p. (intraperitoneal) passages. Leukemia cells from ascites fluid were diluted appropriately in RPMI 1610 medium and injected i.p. (1×10^5^ cells/0.1 mL/mouse) into recipient mice. A daily oral administration of TAC (38 mg/kg via oral gavage) or ACT (25 mg/kg administered i.p.) diluted in PBS was given to leukemic BDF1 mice starting 24 hours from the inoculation of L1210 cells for a total of 5 days. Compounds were prepared fresh daily. A group of untreated leukemic mice received sterile water for injections and served as controls. 

### 2.10. TAC Toxicology

The in vivo toxicity of TAC was evaluated in CD-1 mice using 3 groups of 3 male and 3 female CD-1 mice per group. CD-1 is the classical mouse strain chosen for toxicology studies including safety and efficacy. On Days 1–5 and Days 8–12, animals were administered vehicle (sterile water for injection, Group 1), 38 mg/kg/day TAC (Group 2), or 76 mg/kg/day TAC (Group 3) via oral gavage. Animals were not dosed on Days 6 and 7 and were euthanized on Day 13 for necropsy. Doses were prepared fresh daily from the drug stock. Criteria for evaluation included clinical observations, body weights, limited serum chemistry and hematology parameters, and histopathology evaluation.

## 3. Results

### 3.1. Synthesis and In Vitro Characterization of Triacetyl-5-Azacitidine

**Figure 1:**
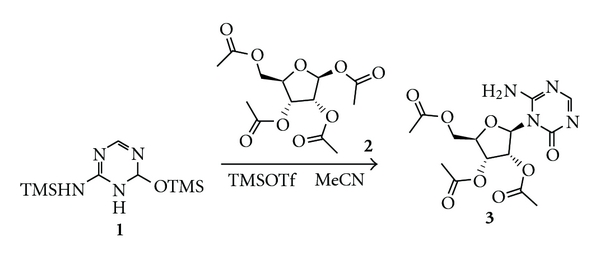
Chemical synthesis of 2′,3′,5′-triacetyl-5-Azacytidine. Results of analytical 1H NMR (CDCl3, 500 MHz): d 8.18(s, 1H), 7.69(s, 1H), 6.33(s, 1H), 5.82(d, 1H, *J* = 3.0 Hz), 5.54(t, 1H, *J* = 4.0 Hz), 5.41(t, 1H, *J* = 6.0 Hz), 4.30–4.41(m, 3H), 2.11(s, 3H), 2.10(s, 3H), and 2.08(s, 3H) ppm. 13C NMR (CDCl3, 125 MHz): d 170.3, 169.6, 169.5, 166.0, 156.0, 153.1, 89.7, 79.9, 73.7, 69.9, 62.8, 20.7, 20.4, and 20.3 ppm.

2′,3′,5′-triacetyl-5-azacitidine has been successfully synthesized through condensation of trimethylsilylated-5-azacytosine and 1,2,3,5-tetra-O-acetyl-*β*-D-ribofuranose (Figure 1). An HPLC detection assay was optimized for TAC and ACT as well as some potential analytes.

The solubility of TAC in comparison to ACT was evaluated at various pH. TAC and ACT were equally soluble at pH1 (40 mg/mL) and pH3 (25 mg/mL), while TAC was more soluble than ACT at pH5 (30 mg/mL versus 15 mg/mL) and pH7 (30 mg/mL versus 10 mg/mL). The stability of the prodrug across a range of pHs is indicated in Table 1. TAC shows remarkable stability at pH 3, 5, and 7.4 with no degradation to the parent compound and 30% hydrolysis to the mono- and diesters only at pH 7.4 after a 29 hr incubation at 25°C. At pH 1, we observed a moderate hydrolysis of TAC (26%) to the mono- and diesters in the first 3 hours of incubation increasing to 60% after 6 hrs. However, after 24, 99% of TAC was hydrolyzed, including 46% to the parent compound, ACT.

A similar experiment to establish the stability of azacitidine in solution resulted in significant degradation of the nucleoside analog throughout the pH range with maximum stability at pH 1. As previously determined, [25] the initial degradation to N-formylguanylribosylurea followed by the irreversible formation of guanylribosylurea resulted in two derivatives with no pharmacological activity (data not shown).

### 3.2. LogP Determination

The log*P* value of a compound is a well established measure of a compound's hydrophilicity. The theoretical physical-chemical properties of TAC were evaluated by measuring the partition coefficient of the un-ionized molecules in two immiscible phases of water and octanol. Azacitidine resulted in an experimental Log*P* value of −2.32 compared to the calculated cLog*P* values of −2.17 while the changes to the molecule by acetylation resulted in an improved Log*P* value for TAC of −0.61 versus a cLog*P* of −0.85.

### 3.3. Permeability across Caco-2 Monolayers

The human colon carcinoma Caco-2 cell model was used to evaluate the oral absorption of the prodrug across membranes and determine the permeability coefficient (absorption rate constant). A sigmoidal relationship exists between drug absorption rates as measured with the in vitro Caco-2 model and human absorption [26]. Permeability coefficients typically range from 5 × 10^−8^ to 5 × 10^−5^ cm/s. Drugs that are well absorbed in humans have permeability coefficients greater than 1 × 10^−6^ cm/s, while drugs and peptides that are absorbed to less than 1% have permeability coefficients less than 1 × 10^−7^ cm/s [27, 28]. Between these ranges, compounds are considered to have intermediate absorption rates. In these studies, ACT was determined to have a permeability coefficient between 1–3 × 10^−6^, compared to 5–7 × 10^−6^ for TAC. This indicates a potential 2-3 fold improvement in intestinal absorption. The efflux ratio is defined as the quotient of the secretory permeability and the absorptive permeability. The efflux ratio was near 1.0, indicating these compounds are not being actively effluxed. No hydrolysis of the prodrug to azacitidine was observed during the course of this experiment.

### 3.4. Pharmacokinetics and Metabolism of TAC in Mice

**Figure 2:**
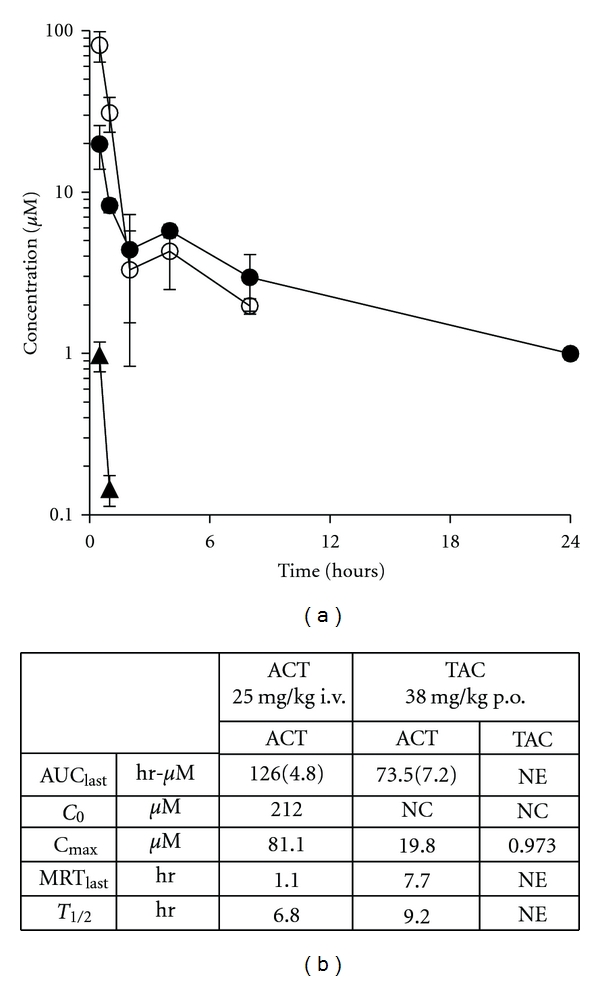
Pharmacokinetics of oral TAC versus i.v. 5-azacitidine in C57BL/6 mice. (a) Mean plasma concentration versus time for tri-acetyl azacytidine (TAC) (▲) and resultant ACT (●) in nontumor bearing mice after a 38 mg/kg dose of oral TAC. Mean plasma concentration versus time for ACT in nontumor bearing mice after 25 mg/kg ACT i.v. (°). Error bars indicate standard deviation. (b) Noncompartmental plasma pharmacokinetic parameters for azacitidine (ACT) and tri-acetyl azacitidine (TAC). AUC_last_ values are mean (standard error). NE—not estimated. NC—not calculated.

TAC was administered at 38 mg/kg to C57/BL6 mice via oral gavage, an equimolar dose equivalent to 25 mg/kg of azacitidine. TAC and its active metabolite ACT were detectable in plasma at 30 minutes after the oral administration (Figure 2(a)). TAC appeared to be rapidly deacetylated leading to a minimal accumulation of the prodrug resulting in plasma concentrations below the limit of quantitation (10 ng/mL) at time points beyond 1 hour after administration. TAC-derived azacitidine reached a peak concentration of approximately 5,000 ng/mL (~20 *μ*M) at 30 minutes with a pharmacologically relevant concentration of at least 0.5 *μ*M being sustained for 24 hours after oral TAC dosing. The mean TAC and TAC-derived ACT plasma concentrations versus time plots after oral administration of TAC prodrug are depicted in Figure 2(a) with the pharmacokinetic parameters reported in Figure 2(b). ACT was also administered i.v. as a control to emulate the standard means of administration in the clinical setting. The T_1/2_ and MRT_last_ values for TAC-derived azacitidine were 9.2 hours and 7.7 hours, respectively, whereas i.v. administration of ACT resulted in T_1/2_ and MRT_last_ values of 6.8 hours and 1.1 hours, respectively. The AUC_last_ values for plasma azacitidine were 73.5 hr-*μ*M and 126 hr-*μ*M after oral administration of TAC prodrug or i.v. administration of ACT, respectively. While the absolute bioavailability of derived azacitidine after oral TAC administration is 58%, the prolonged T_1/2_ and MRT_last_ values for azacitidine observed in plasma after oral TAC administration suggest a protracted absorption and conversion of the TAC prodrug at the gastrointestinal or presystemic level (Figure 2(b)). The log-linear regression analysis of ACT concentration data through 2 hours revealed an increased T_1/2*α*_ value for ACT after p.o. TAC administration compared with i.v. ACT administration (0.73 hr versus 0.32 hr, resp.), also suggesting protracted absorption and both presystemic and systemic conversion of TAC.

### 3.5. Pharmacodynamic Effect of Triacetyl-5-Azacitidine

MTS assays (Promega, Madison, Wis, USA) were performed to determine the in vitro effects of ACT and TAC on L1210 cell proliferation. TAC had no effect on L1210 cell viability even at doses 100-fold higher than the known IC50 of ACT (0.2 uM) [29], and this is an indication that this compound does not get activated in vitro. This result was predicted, since cells in culture lack the necessary esterase activity to convert the prodrug to ACT that remains below the limit of detection using our LC-MS method.

Gene-specific methylation PCR was performed in order to confirm whether TAC could affect the methylation level of a specific target even in the absence of cellular toxicity. ACT, which is the active derivative of TAC, has been extensively studied by numerous research groups to determine specific cellular methylation targets and mechanism of action [30, 31]. P15INK4B is a classic target known to be hypermethylated in AML and MDS and attributes to a poor prognosis in those patients [32, 33]. 0.1 uM and 10 uM ACT decreased P15 promoter methylation by 39% (±3) and 49% (±17), respectively. In comparison, 1 uM and 100 uM TAC decreased P15 promoter methylation by only 19% (±8) and 23% (±16), respectively.

**Figure 3:**
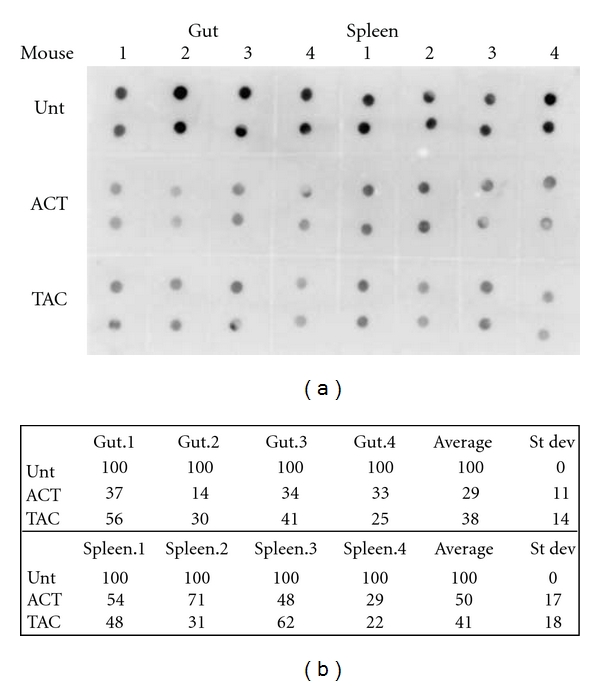
Effect of DNMT inhibitors on global DNA methylation in tissues of mice exposed for 5 days to ACT or TAC. (a) Dot blot of mouse tissues isolated 24 hours after treatment with 25 mg/kg i.v. ACT or 38 mg/kg p.o. TAC. Groups were compared to untreated animals (unt). (b) Densitometry of methylation levels represented in (a).

In an in vivo experiment designed to evaluate the effect of a prolonged administration of TAC on global methylation, TAC (38 mg/kg p.o.) or ACT (25 mg/kg i.p.) diluted in PBS was administered to C57BL6 mice every 24 hours for 5 consecutive days (four animals per group). Tissue methylation levels are reported in Figure 3. A significant reduction in global DNA methylation was detected in the gut and spleen after the administration of TAC, comparable to the methylation decrease detected after the administration of ACT. We did not observe a significant change in global methylation status in kidney or liver tissue (data not shown). The positive results in gut and spleen were confirmed using the EpiGentek's Methylamp Global DNA Methylation Quantification Kit. This quantitative analysis confirmed a comparable 50%–60% decrease in global methylation by both TAC and ACT (data not shown).

### 3.6. Antileukemic Activity of Triacetyl-5-Azacitidine

**Figure 4:**
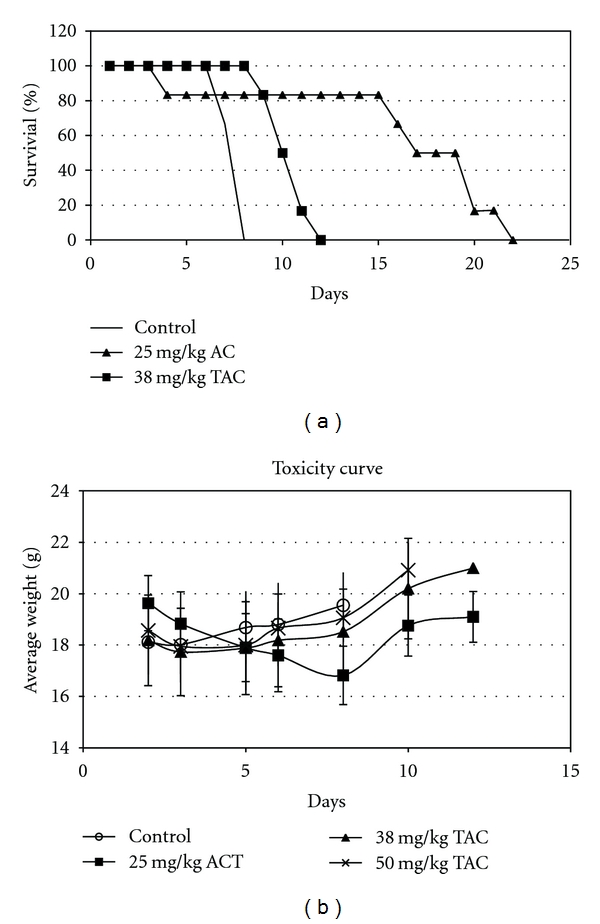
(a) Antileukemic activity of TAC and ACT in BDF1 mice bearing L1210 lymphocytic leukemia cells after 5 days of treatment with indicated concentrations of oral TAC or i.p. ACT. (b) Toxic effect as indicated by average weight loss of the same tumor bearing mice.

The L1210 lymphocytic leukemia model was used to determine the efficacy of TAC in an animal model. The L1210 model is a classical model used to predict for compounds that are effective against leukemias and lymphomas. A daily oral administration of TAC (38 mg/kg equimolar to 25 mg/kg of ACT and a higher dose of 50 mg/kg) or azacitidine i.p. (25 mg/kg) was given to leukemic mice for a total of 5 days. The median survival time for the untreated control group was 8 days, while the median survival time for the TAC treated groups was 12 days resulting in a 50% increased lifespan, demonstrating not only an epigenetic modulating effect, but also antileukemic activity. Azacitidine at the maximum tolerated dose of 25 mg/kg administered i.p. resulted in a 17-day survival with a 112% increased lifespan (Figure 4(a)). The toxicity curve (Figure 4(b)) indicates a nonsignificant weight loss for the TAC-treated groups at day 3 followed by a rapid recovery indicating the lack of toxicity of this prodrug coupled with a significant antileukemic effect, while the azacitidine-treated animal showed a 16% weight reduction with a significant recovery once the treatment was completed.

### 3.7. TAC Toxicology

The in vivo toxicity of TAC was evaluated by orally administering repeated doses to CD-1 mice for two weeks. On Days 1–5 and Days 8–12, animals were administered vehicle, 38 mg/kg/day TAC (Group 2, equivalent to 25 mg/kg of ACT), or 76 mg/kg/day TAC (Group 3) via oral gavage. Criteria for evaluation included clinical observations, body weights, limited serum chemistry and hematology parameters, and histopathology evaluation of a limited number of tissues from vehicle-treated and 76 mg/kg/day TAC-treated animals.

There were no deaths and no notable TAC-associated body weight changes during the study. Additionally, there were no TAC-associated changes in liver or renal function markers, including alanine and aspartate aminotransferases, alkaline phosphatase, bilirubin, blood urea nitrogen, and creatinine.

**Figure 5:**
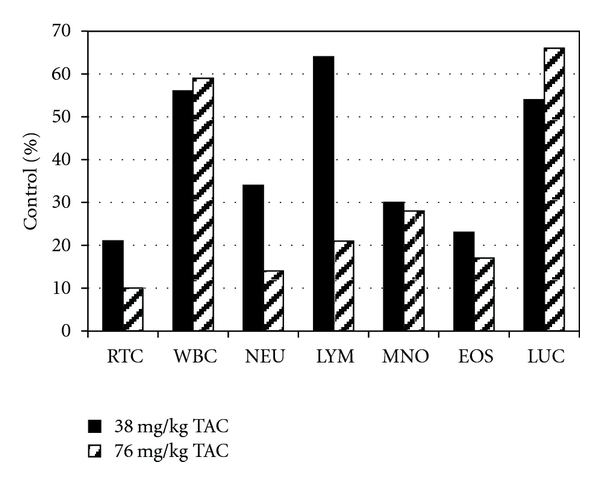
TAC-associated effect on hematological parameters following TAC administration to CD-1 mice. RTC: reticulocyte count, WBC: white blood cell count, NEU: neutrophil count, LYM: lymphocyte count, MNO: monocyte count, EOS: eosinophil count, LUC: large unstained cell count.

TAC-associated changes in various hematological parameters were observed (Figure 5). This included a reduction in white blood cell counts, absolute lymphocytes, and absolute leukocytes by 41%, 31%, and 24%, respectively, for the 38 mg/kg/day dosage group. The reduction values for the 76 mg/kg/day group only slightly increased by 44%, 31%, and 34%, respectively. There were TAC-related findings in the bone marrow (hypocellularity) and in the lymph nodes (inactive germinal centers) which reflect the decreased overall white blood cell count reductions. An additional microscopic TAC-related finding was duodenum apoptosis of the crypt epithelium in the 76 mg/kg/day-treated animals. Other observations included a decrease in myeloid and erythroid compartment cellularity correlating with microscopic findings in various tissues.

## 4. Discussion

Epigenetic modulators are appealing cancer therapeutics due to their potential reversibility and preference for highly proliferating cancer cells. Azacitidine and decitabine have demonstrated improved clinical success in hematologic malignancies with longer or continuous infusion [34]. However, the aza-pyrimidine ring is unstable in aqueous solutions and has poor bioavailability, limiting the use of oral formulations that would be more convenient for the patients, help in long-term dosing, and reduce local side effects when administered as subcutaneous depot.

We have successfully synthesized an azacitidine prodrug, 2′,3′,5′-triacetyl-5-azacitidine and shown its favorable physical-chemical characteristics. TAC has demonstrated higher solubility and stability in vitro across a wide pH range compared to its parent compound azacitidine, which is of great benefit for avoiding rapid degradation in the acidic environment of the stomach and the more neutral environment of the periphery. TAC has also shown a more favorable log*P*. While Log*P* values may not always be a completely accurate determination of the lipophilicity of ionizable compounds, the data confirm the efficient drug absorption at the gastrointestinal level and bioavailability observed.

In vivo analyses were performed comparing the desired oral version of the prodrug with the classical administration route of ACT (i.v. or subcutaneous). The stability and bioavailability of the prodrug was confirmed in vivo, where oral TAC-derived azacitidine had a terminal half-life of 9.2 hours versus 6.8 hours for i.v. administered azacitidine. Additionally, the alpha phase half-life (T_1/2*α*_) value after oral TAC administration was longer compared with i.v. ACT—0.73 hr versus 0.32 hr, respectively. The half-life values indicate a protracted absorption of the nucleoside prodrug at the gastrointestinal level and an effective conversion by serum esterases. This may allow maintenance of ACT concentrations over a 24-hour period as opposed to roughly 6 hours for i.v. ACT. The half-life of azacitidine in this murine model is longer in our study compared to some of the previous literature [35, 36]. This is likely due to an improved sensitivity of our analytical methodology that allows monitoring the concentration of azacitidine below 100 ng/mL.

TAC also demonstrated a pharmacodynamic effect in vivo. Oral TAC appears comparable to i.v. AC in its ability to suppress global methylation in normal tissues. Further analysis will be necessary to determine if this is a tissue-specific effect or effective in altering the methylation status of cancer cells and to identify specific gene targets. TAC was less effective than ACT at improving life span in the leukemic L1210 animal model, possibly due to a higher C_max⁡_ achieved by i.p. administration compared to oral, confirming the dichotomy between epigenetic therapy that is effective at low extended doses versus the cytotoxic effect achieved with higher doses and linked to a higher C_max⁡_ [37].

Animals administered TAC for 2 weeks resulted in no deaths and no weight loss, indicating minimal general toxicity. However, TAC did induce the expected changes in hematological parameters, bone marrow, and lymph nodes. Gastrointestinal toxicity was observed at the higher TAC dose with duodenum apoptosis of the crypt epithelium. This effect is possibly due to the localized hydrolysis of the prodrug with formation of azacitidine known to cause gastrointestinal toxicities in patients. Several have attempted to overcome the lack of stability and poor bioavailability of the current DNMT inhibitors. Oral formulations of azacitidine and decitabine have not been successful due to their hydrolysis in the aqueous environment of the intestinal tract and rapid deamination. An oral film-coated formulation of azacitidine has been recently evaluated in four human subjects resulting in 17.4% bioavailability for an 80 mg dose with a mean plasma half-life of 0.39 hr versus 0.68 hr for a subcutaneous formulation [38]. A very recent Phase I study of oral azacitidine showed a high interpatient variability in the bioavailability of azacitidine ranging from 6.3% to 20% (33) indicating the need for a more controlled delivery [39].

Other groups have proposed modification of the structure at the N4 position such as the addition of a 2-(p-nitrophenyl)ethoxycarbonyl group (NPEOC) to protect the azacytosine ring [40]. This derivative is similar to the 5-FU prodrug capecitabine. The N4-NPEOC-5-aza-2′-deoxycytidine has been shown to be activated to decitabine resulting in global and gene-specific DNA demethylation in cells expressing carboxylesterase 1 [41]. The NPEOC derivatives are characterized by very low solubility in water and their elevated hydrophobicity could lead to an improved oral bioavailability; however, in vivo data in animal models or humans are not available [40]. Protection of the N4 has also been achieved in sapacitabine by incorporating a N4-palmitoyl group of (1-(2-C-cyano-2-deoxy-*β*-D-arabino-pentafuranosyl) cytosine (CNDAC). This modification has resulted in better diffusion into gastrointestinal cells allowing the oral administration of the drug [42]. A Phase I study of oral sapacitabine confirmed its metabolism to CNDAC; however, it resulted in pharmacokinetic variability possibly due to differences in absorption or metabolism [43].

To improve the stability characteristics of decitabine and increase its intracellular delivery, the nucleoside analog has been incorporated into a guanine dinucleotide to generate S110 (5′-AzapG-3′). S110 has shown stability in aqueous solutions and toxicity comparable to that of decitabine with increased resistance to enzymatic degradation (deamination) before incorporation into DNA following endonuclease and/or phosphodiesterase cleavage [44].

We have synthesized and evaluated an acetylated derivative of azacitidine, 2′,3′,5′-triacetyl-5-azacitidine (TAC) as an oral prodrug. While this is not a totally new approach, it was never utilized for this derivative and in this set of diseases. A few examples exist in the literature of this type of derivatization for the oral delivery of pyrimidine analogs. Triacetyl-6-azauridine was developed to overcome the poor oral absorption of azauridine, an antipsoriasis drug [45]. 2′,3′,5′-tri-O-acetyluridine (PN401) was synthesized as a rescue agent for 5-fluorouracil-based therapies [46, 47].

Taken together, our data indicate the triacetylated derivative of azacitidine may be an effective prodrug that could allow continuous exposure to azacitidine at low doses resulting in a protracted DNA hypomethylation. The superior stability and solubility of TAC, together with its minimal toxicity, will encourage the further investigation of its mechanism of action, its epigenetic modulatory effect, and its ability to slow down the progression of MDS into AML.

## Figures and Tables

**Table 1:** Stability of TAC and its metabolites at various pH.

Sample	TAC (%)	DAC (%)	MAC (%)	ACT (%)
Control	100			

pH 1—1 hr	99	1	0	0
pH 1—3 hr	74	22	4	0
pH 1—6 hr	38	41	21	0
pH 1—29 hr	1	22	31	46

pH 3—1 hr	97	3	0	0
pH 3—3 hr	96	4	0	0
pH 3—6 hr	92	6	2	0
pH 3—29 hr	93	4	2	1

pH 5—1 hr	98	2	0	0
pH 5—3 hr	94	6	0	0
pH 5—6 hr	98	2	0	0
pH 5—29 hr	94	4	1	1

pH 7.4—1 hr	96	4	0	0
pH 7.4—3 hr	91	7	2	0
pH 7.4—6 hr	85	12	3	0
pH 7.4—29 hr	69	17	13	1

TAC: tri-acetyl-azacitidine; DAC: di-acetyl-azacitidine; MAC: mono-acetyl-azacitidine; ACT: azacitidine.
